# Validity of a three-item dating abuse victimization screening tool in a 11–21 year old sample

**DOI:** 10.1186/s12887-022-03397-w

**Published:** 2022-06-10

**Authors:** Emily F. Rothman, Julia K. Campbell, Ariel M. Hoch, Megan Bair-Merritt, Carlos A. Cuevas, Bruce Taylor, Elizabeth A. Mumford

**Affiliations:** 1 Department of Occupational Therapy, Boston University, 635 Commonwealth Ave, Boston, MA 02215 USA; 2 Department of Health Behavior, Gillings School of Global Public Health, University of North Carolina at Chapel Hill, Chapel Hill, NC USA; 3 Boston University School of Medicine, Boston, MA USA; 4 Northeastern University, Boston, MA USA; 5 NORC at the University of Chicago, Chicago, IL USA

**Keywords:** Dating abuse, Adolescent health, Intimate partner violence, Screening tool

## Abstract

**Background:**

Dating abuse (DA) is prevalent and consequential, but no brief DA screening tools are available for use in pediatric or other settings. This study was designed to determine the sensitivity, specificity, and predictive values of the MARSHA-C, which is a three-item DA victimization screening tool.

**Methods:**

The participants were 224 U.S. youth ages 11–21 years old (20% male, 77% female, 3% non-binary gender). Youth completed an online questionnaire about adolescent relationship abuse. The survey included the Measure of Adolescent Relationship Harassment and Abuse (MARSHA), which is a comprehensive DA measurement instrument normed on a nationally representative sample. Of 34 DA victimization items from the MARSHA, the three most prevalent items were hypothesized to have good predictive validity of the full scale score as a brief, screening version (MARSHA-C). The sensitivity, specificity, positive predictive value, and negative predictive value of the MARSHA-C to identify victims of DA was calculated.

**Results:**

Using the MARSHA as the reference standard, the cutpoint of 1 on the MARSHA-C screening tool was identified as optimal. The MARSHA-C had a sensitivity of 84%, a specificity of 91%, and positive predictive value of 91%. Thus, for youth who endorse ≥ 1 MARSHA-C items, there is a 91% probability that they have experienced DA in the past year. Exploratory analyses by demographic subgroups suggest that the predictive validity of the MARSHA-C is approximately equivalent for females and males, younger and older adolescents, Asian, Black, Latinx, Multiracial and White youth, and heterosexual and lesbian, gay, and bisexual youth.

**Conclusions:**

The MARSHA-C can be used to detect DA among 11–21-year-old youth via online surveys for research purposes, or in clinical care settings to facilitate proactive patient counseling or parent-oriented anticipatory guidance.

## Background

Dating abuse (DA) is a prevalent and consequential public health problem. In the U.S., approximately 1 in 6 girls and 1 in 12 boys who attend high school and have dated report having experienced physical and/or sexual assault by a dating partner in the past year [1]. Adolescents who experience DA are at increased risk for a range of physical and mental health problems including depression, anxiety, post-traumatic stress disorder symptoms, self-harm, disordered eating, sexually transmitted infections, unplanned pregnancy, academic problems, injuries, and death [2–6]. Longitudinal studies have also established that those who are victimized are at increased risk for subsequent victimization in adolescence and adulthood [4, 7, 8].

The U.S. Preventive Service Task Force recommends that clinicians screen women of reproductive age for partner violence victimization, and provide or refer those who screen positive to ongoing support services [9]. In addition, the American Academy of Pediatrics (AAP) calls for primary care-based preventive counseling about healthy dating relationships [10]. In particular, clinicians should screen pediatric patients with so-called “red flags” for DA including a history of sexually transmitted infections [11], pregnancy [12], depression [4, 12], frequent appointment cancellations, or somatic complaints that do not fit the medical history [13]. In such cases, recognizing and addressing the problem may prevent the abuse from getting worse or improve the chances that the patient will make a plan to leave the relationship safely. Research with adult survivors of partner violence has found that patients who talked with a health care provider about the abuse were four times more likely than those who did not talk with a health care provider to use a helping resource [14]. For pediatric patients who screen positive, referrals to local counselors with expertise in DA, and national dating abuse hotline and advocacy services may be helpful [15].

There are several screening tools for identifying adult survivors and perpetrators of partner violence in clinical settings [16, 17]. Some of the most widely-used screening tools for adults include the four-item Hurt, Insult, Threaten, and Scream (HITS) (sensitivity 30–100%, specificity 86–99%), the eight-item Woman Abuse Screen Tool (WAST) (sensitivity 47–93%, specificity 56–96%), and the five-item Abuse Assessment Screen (AAS) (sensitivity 93–94%, specificity 55–99%) [17]. However, these tools were not developed to identify partner abuse in young people and have not been tested with adolescents. Some items from these adult-oriented screeners may not apply to adolescent dating relationships. For example, this item from the AAS would less commonly be relevant for adolescents: “Since you have been pregnant, have you been hit, slapped, kicked, or otherwise physically hurt by someone?” The HITS, WAST, and AAS were also developed two decades ago, and do not assess technology-facilitated partner abuse, which is prevalent among adolescents [18].

To date, there have been no sensitivity and specificity determination studies to establish the utility of diagnostic screeners for youth DA in clinical settings. The present study validated a three-item test, the MARSHA-C, that can be used in clinical settings to detect DA victimization in pediatric patients, or other settings where large numbers of youth are found (e.g., schools or online settings). The aim of the present study was to determine the sensitivity, specificity, and predictive values of the MARSHA-C.

## Methods

### Participants

This cross-sectional survey study was approved by the Institutional Review Board (IRB) at the first author’s institution. Although this study was originally planned to take place in a pediatric clinical setting in person, pediatric care in the U.S. in 2020 was often provided online due to the COVID-19 pandemic. As such, this research also pivoted to online data collection with the idea that results could inform both in-person and online clinical encounters in the future. Subjects were recruited May-December 2020 through flyers posted in the pediatric emergency department at a hospital in a large urban area in the northeast U.S. and through social media. Flyers and online advertisements were in English and informed viewers that youth ages 11–21 years old with dating experience had the opportunity to participate in an online survey that would take 10 min. A link to the survey was provided. Interested youth who clicked the link landed on the eligibility survey. Eligible youth were those 11–21 years old, who could read and write in English, lived in the U.S., and reported that they were dating, hooking up, or in a romantic relationship in the past year. Eligible youth then viewed a consent statement (or an assent statement if they were younger than 18 years old). Those who assented/consented advanced to the survey. Parental consent was not required because risks to youth were low, given that the survey was anonymous and participants could skip any questions that they did not want to answer [19, 20].

**Fig. 1:**
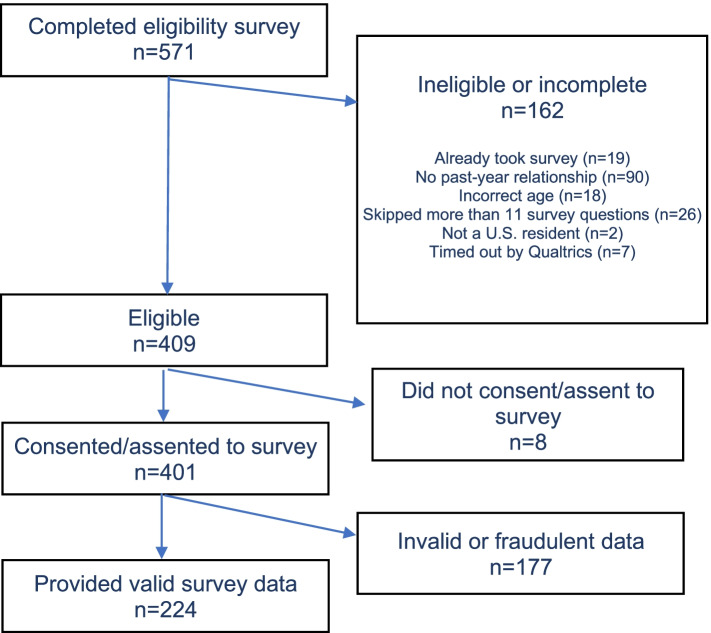
Participant recruitment

We received 571 responses to the online, self-administered eligibility screening survey (Fig. 1). Approximately 72% (*n* = 409) were eligible entries. The 162 ineligible entries were ineligible for the following reasons: 19 had already completed the survey, 90 had no past-year dating relationship, 18 were too young or too old, 26 skipped > 10 questions of the survey, 2 were not US residents, and 7 timed out while attempting to complete the survey. Of the eligible entries, 99% (*n*= 401) consented to participate and completed surveys. Of the 401 completed surveys, and in keeping with other surveys using social media recruitment [21], 49% of responses (*n*= 177) were flagged as potentially fraudulent (e.g., from a bot) because one or more of the following were true: (a) the zip code did not match the state of the U.S. where the participant reported that they lived; (b) all five of the open-ended questions, which required text to be entered, were blank or contained nonsense strings; (c) the respondent’s IP address was outside the U.S; (d) the same respondent answered the survey multiple times; or (e) the latitude and longitude of the IP address did not match the state and/or zip code where the participant reported that they lived. Social media survey recruitment can generate samples where 95% of responses are fraudulent [21], which is why it is important to use fraud detection procedures [22]. All potentially fraudulent responses were removed from the dataset. The final analytic sample was *n* = 224 (Fig. 1).

The majority of participants (79%) were 17–21 years old, and the mean age was 18.6 (SD 2.3) years. The sample was 77% female, 20% male, and 3% non-binary or other gender, 41% White, 20% Asian, 18% Hispanic or Latinx, 12% Black or African American, 7% Multiracial and 3% other race (Table 1).

**Table 1:** Demographics of sample (*N* = 224)

	Full sample %(n)	Dating abuse survivors %(n)	Chi-sq., *p*-value
Total	100% (224)	100% (96)	–
Age			0.02, *p* = 0.88
11–16 years old	21.4% (48)	21.9% (21)	
17–21 years old	78.6% (176)	78.1% (75)	
Gender			0.74, *p* = 0.69
Male	20.1% (45)	18.8% (18)	
Female	76.8% (172)	77.1% (74)	
Transgender, non-binary, intersex, gender-queer or other	3.1% (7)	4.2% (4)	
Race/ethnicity			5.19, *p* = 0.39
Asian	19.5% (42)	17.8% (16)	
Black or African American	11.6% (25)	13.3% (12)	
Hispanic or Latinx	18.1% (39)	15.6% (14)	
White	40.5% (87)	44.4% (40)	
Multiracial	7.4% (16)	4.4% (4)	
Other race	2.8% (6)	4.4% (4)	
Sexual orientation			.458, *p* = 0.50
Gay, lesbian or bisexual	29.5% (66)	27.1% (26)	
Heterosexual	70.5% (158)	72.9% (70)	
Asexual	0% (0)	0% (0)	
State of residence			1.08, *p* = 0.78
Northeast	65.3% (145)	64.6% (62)	
Midwest	3.6% (8)	4.2% (4)	
South	17.6% (39)	15.6% (15)	
West	13.5% (30)	15.6% (15)	
Relationship status			0.55, *p* = 0.76
Single, never married	79.4% (173)	81.7% (76)	
Living with someone in a committed relationship	9.6% (21)	8.6% (8)	
Other	11.0% (24)	9.7% (9)	

### Procedures

Participating youth completed an 83-item online survey. On average, it took youth 10 min to complete it. After completing the survey, participants were directed to a separate survey that collected their email address via which they received a $5 Amazon.com gift card as remuneration.

### Measures

#### Reference standard: The MARSHA

The Measure of Adolescent Relationship Harassment and Abuse (MARSHA) is a comprehensive DA measurement instrument that includes items on past year physical, sexual, and psychological adolescent DA, as well as technology-facilitated DA, social control, and invasion of privacy. The full instrument, described elsewhere, is psychometrically sound, was normed on a nationally representative U.S. sample of 11–21-year-old youth in 2019, and comprises 34 victimization items [23]. In the present study, the full version of the MARSHA was used as the gold standard. The MARSHA is prefaced with the following instruction: “Think about all of the people you were dating, hooking up with or in a romantic relationship with in the past year. Answer the following questions thinking about these people. Did the following things happen? (Do not count times when these things happened for fun or as a joke).” Response options for each item are on a 4-point likert-type scale denoting the number of times each abusive behavior has occurred in the past year; 0 times, 1–3 times, 4–10 times, or more than 10 times. Cronbach’s alpha for internal consistency of the full MARSHA’s five victimization subscales (i.e., privacy control, social control, physical abuse, sexual abuse, and intimidation) ranged from α = 0.79 to α = 0.90 in a sample of *N*= 1,257 11–21-year-old youth. In the present sample, the full MARSHA victimization scale reliability was α = 0.90.

The MARSHA is a continuous scale and does not have a recommended cutoff for determining the binary presence or absence of DA victimization. A score of 6 meant that the participant had experienced a minimum of 5 acts of DA in the past year. For the present study, we classified those with a score ≥ 6 as DA survivors (*i.e*., DA-positive), and those with no DA experience or infrequent experience as DA-negative.

#### The screening test: the MARSHA-C

The acronym MARSHA-C denotes the clinical screening version of the victimization questions of the MARSHA. The MARSHA-C items were selected from the full MARSHA instrument victimization questions by reviewing which items were most commonly endorsed. The three MARSHA-C questions are: “They yelled, screamed or swore at me,” “They asked or pressured me for a nude or almost nude photo or video of me, when I did not want to give them one,” and “They made me feel like I could not break up with them or get out of the relationship.” For each of these three acts that a respondent endorsed, they received one point, regardless of how frequently they had experienced each act. This is because the clinical screening tool version of the MARSHA-C is designed to be a series of three yes/no questions. Thus, total scores on the MARSHA-C ranged from zero to three. A goal of the present study was to determine the best cutpoint on the MARSHA-C to indicate the presence of DA.

### Demographic variables

Youth were asked several demographic questions in order to characterize the sample. Participants were asked to report their age in years, and those 11–16 years old were classified as younger, while those 17–21 years old were classified as older. Race and ethnicity were collected through a single item that asked, “How do you describe your race and ethnicity?” Participants could select one or more of seven response options. To determine sexual orientation, youth were asked about their attractions and about their dating experience. Those reporting past year same sex attraction, or same sex dating, were classified as gay, lesbian or bisexual.

### Statistical analysis

#### Determining sample size for adequate sensitivity and specificity analysis

We used the following formula to determines the number of cases needed to estimate sensitivity [24]:

$${\mathrm{n}}_{\mathrm{cases}}=\frac{{\mathrm{Z}}^{2}\widehat{\mathrm{P}}\left(1-\widehat{\mathrm{P}}\right)}{{\mathrm{d}}^{2}}$$

In this formula, Z^2^ was set at 1.96 to represent an α = 0.05, the $\widehat{\mathrm{P}}$ represents the value of sensitivity (0.85), and d represents the maximum marginal error of 0.10 (*NB*: d^2^ = 0.01). Solving for n_cases_ we calculated: 3.8416 × 0.85 × 0.15 = 0.489804 / 0.01 = 49 cases. The total sample size required is the total number of cases divided by the prevalence of the condition in the population, which was 40.9%. This yielded a total sample size requirement of 119, which we exceeded with *N* = 224 participants.

**Fig. 2:**
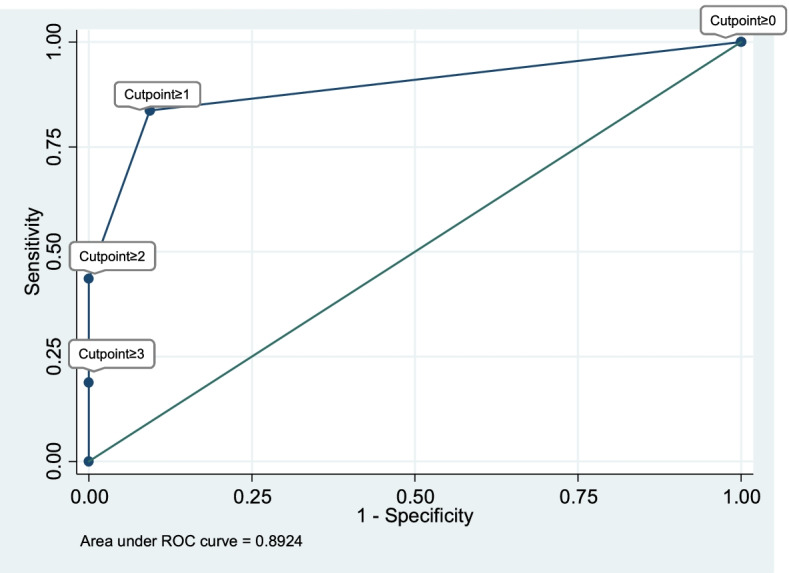
ROC curve analysis for the MARSHA-C

The rates of DA were also calculated with 95% confidence intervals for the MARSHA-C using cutpoints of 1, 2 or 3 (see Table 2). A receiver operator characteristic (ROC) curve was created by plotting the sensitivity of each MARSHA-C cutpoint against the false positive rate (100-specificity, Fig. 2). The area under the curve (AUC) was used to determine the optimal cutpoint for MARSHA-C. In general, AUCs of 0.7–0.8 are considered acceptable, while AUCs of 0.8–0.9 are considered excellent [25]. The closer that a ROC curve gets to the top left-hand corner of the graph, which represents a combination of 100% sensitivity and 100% specificity, the better the cutpoint [26]. After determining the optimal MARSHA-C cutpoint, we calculated the sensitivity, specificity, ROC area, positive predictive value, negative predictive value, and accuracy of the MARSHA-C for the entire sample (Table 3). We also explored whether there was variation in sensitivity, specificity, and accuracy for demographic subgroups (Table 3).

**Table 2:** The sensitivity, specificity, positive predictive value, and negative predictive value with 95% confidence intervals of MARSHA-C at different cut off scores

MARSHA-C cut off score	% of study sample	Sensitivity (95% CI)	Specificity (95% CI)	Positive predictive value (95% CI)	Negative predictive value (95% CI)
1	48.2%	83.8% (75.8%-89.9%)	90.7% (83.5%-95.4%)	90.7% (83.6%-95.5%)	83.6% (75.6%-89.8%)
2	22.8%	43.6% (34.4%-53.1%)	100% (96.6%-100.0%)	100% (93.0%-100.0%)	61.8% (54.2%-69.1%)
3	9.8%	18.8% (12.2%-27.1%)	100.0% (96.6%-100.0%)	100.0% (84.6%-100.0%)	53.0% (45.8%-60.0%)

**Table 3:** Sensitivity, specificity, and predictive value of the MARSHA-C for detecting dating abuse victimization in adolescents ages 11–21 years old, using a cutpoint of 1 on the MARSHA-C

	Sample size	Prevalence	Sensitivity (%)	Specificity (%)	ROC area	Positive Predictive Value (%)	Negative Predictive Value (%)	Overall Accuracy^a^
Full sample	224	48.2%	83.8%	75.8%	0.872	90.7%	83.6%	87.1%
Female	172	48.3%	81.5%	90.0%	0.858	90.4%	80.9%	85.5%
Male	45	44.4%	90.5%	95.8%	0.932	95.0%	92.0%	93.3%
Young (11–16)	48	47.9%	76.0%	82.6%	0.793	82.6%	76.0%	79.2%
Older (17–21)	176	48.3%	85.9%	92.9%	0.894	92.9%	85.7%	89.2%
Heterosexual	158	50.6%	84.1%	91.4%	0.878	92.5%	82.1%	87.3%
Lesbian, gay, bisexual	66	42.4%	82.8%	89.2%	0.860	85.7%	86.8%	86.4%
Asian	42	47.6%	85.0%	86.4%	0.857	85.0%	86.4%	85.7%
Black	25	56.0%	81.3%	88.9%	0.851	92.9%	72.7%	84.0%
Hispanic or Latinx	39	30.8%	70.6%	100.0%	0.853	100.0%	81.5%	87.2%
White	87	55.1%	89.8%	89.5%	0.896	91.7%	87.2%	89.7%
Multiracial	16	31.3%	75.0%	83.3%	0.792	60.0%	90.9%	81.3%

^a^Accuracy is the number of true positives (TP) and true negatives (TN) divided by sample size (n), or (TP + TN/n)

## Results

The full version of the MARSHA identified 117 cases of DA victimization in the sample (52.2% prevalence). There were no significant differences in the demographic characteristics of those who were identified as DA victims as compared to those in the full sample (Table 1). In this sample, using the full version of the MARSHA, 17% reported one or more experiences of physical DA victimization, 25% reported being forced to do something sexual that they did not want to do or that they experienced a sexual violation such as having a nude photo shared non-consensually, 35% experienced emotional abuse, and 43% reported technology-facilitated DA. For example, 22% of the sample reported that their dating partner had used social media or other apps to keep track of them and monitor where they were going or where they had been, and 22% reported that their dating partner looked through their phone or other device when they did not want them to do that.

The three items selected for use as the MARSHA-C were the most prevalent MARSHA victimization items, with one exception. The item “they stopped talking to me and I felt punished, hurt, or scared,” was endorsed by 38% of the sample but was not considered for inclusion on the MARSHA-C because in some cases ceasing contact may be a healthy behavior. The next three most prevalent items were included on the MARSHA-C: approximately 28% of the sample reported that someone had asked or pressured them for a nude or almost nude photo or video when they did not want to give one, 28% reported that a partner yelled, screamed, or swore at them, and 25% reported that a partner made them feel like they could not break up or get out of the relationship.

Table 2 provides the sensitivity, specificity, positive predictive value, negative predictive value, likelihood ratios and post-test odds of DA using different cutpoints of MARSHA-C to identify survivors of DA. The receiver operator characteristic (ROC) curve (Fig. 2), and the data presented in Table 2, demonstrate that a MARSHA-C score of 1 was selected as the optimal cutpoint for detecting DA experience. Visual inspection of the ROC indicates that the cutpoint closest to the upper left corner, or the point that includes the largest AUC, is the cutpoint ≥ 1. This cutpoint has a sensitivity of 83.8% and a specificity of 90.7%.

Using a MARSHA-C cutpoint of 1, we investigated accuracy of the test for the overall sample (Table 3). Next, on an exploratory basis, we investigated sensitivity, specificity, and accuracy by subgroups of interest, including by gender, age, sexual orientation and race (Table 3). Subgroup analyses for females (*n* = 169), older adolescents (*n* = 172), and heterosexual youth (*n* = 155) were sufficiently powered to be considered non-exploratory, whereas subgroup analyses by racial subgroup, on males, younger adolescents and lesbian, gay and bisexual youth were exploratory. The subgroup analyses found that sensitivity, specificity, and accuracy did not vary widely by demographic subgroup.

The accuracy of a screening test is the number of true positives and true negatives divided by the sample size. Using the whole sample, the MARSHA-C had an accuracy of 87%. Accuracy was approximately equivalent for females and males, and heterosexual and sexual orientation minority youth (Table 3).

## Discussion

The three MARSHA-C DA victimization screening questions may be used to identify youth with experiences of past-year DA. Being asked to complete a self-report version of the MARSHA-C prior to health care appointments may help providers identify youth in need of help related to DA experiences and may encourage DA-experienced youth to communicate with their pediatricians about their safety-related needs. The estimated sensitivity and specificity of the MARSHA-C (84% and 91%, respectively) suggest it is an effective short tool for samples of 11–21-year-old youth. The sensitivity and specificity of the MARSHA-C is on par with widely-used adult partner violence screening tools [17]. For any patient who responds with one or more yes answers, there is a 91% probability that the patient has experienced DA victimization in the past year (positive predictive value) and they are 9 times more likely to have experienced DA in the past year than someone with a MARSHA-C score of 0 (likelihood ratio of a positive result). A strength of the MARSHA-C is that the specificity is high (91%), reducing the likelihood that a youth will be incorrectly identified as a DA survivor when they have not experienced it, which is important because there can be stigma associated with being identified as a DA survivor.

Adolescents who are experiencing DA are generally reluctant to seek help from adults [27, 28], although research suggests the majority (89%) will confide in a friend, and 40% may talk to a sibling or cousin [28]. The problem with confiding in adolescent peers is that they do not always offer good advice or know how to help. Clinicians may be able to offer more meaningful solutions for youth who are experiencing abuse in relationships, and the MARSHA-C is a brief screening tool that may allow them to broach the conversation. Adolescents report that their doctors are trusted sources of information about dating relationships and that they would like pediatricians to ask them about DA [29]. The MARSHA-C could be integrated into other written or tablet-based questionnaires that youth are given at annual well visits, or the three items could be used as a stand-alone screener. Best practices for communicating with patients about DA are provided by Randell and Ragavan (2019), who recommend validating the patient after the disclosure, making a warm handoff to supportive resources, partnering with community-based agencies with expertise in the topic, remaining non-judgmental when patients decline supportive services, not documenting the abuse in medical records due to the risk of others seeing the record at a future point, and safety planning with the patient [15]. Future research should investigate how pediatric and adolescent medicine practices choose to incorporate the MARSHA-C, whether MARSHA-C data are included in electronic medical records, and whether or not positive screens result in referrals to helping resources. 

Limitations of this study include the fact that the study was conducted online rather than in person, and some clinicians may prefer to pose questions about DA aloud to patients. That possibility notwithstanding, it is possible that youth were more likely to report DA experiences truthfully via an online survey than they would be if asked aloud by a provider in a clinical setting. Some health care providers may choose to request that patients complete screening surveys online prior to office visits, and the MARSHA-C may be useful for that purpose. A second limitation is that the reference standard, the MARSHA, is scored continuously. We conservatively used a cutpoint of > 5, which meant that youth with a minimum of 5 experiences of DA in the past year were classified as survivors. If we had selected a lower cutpoint on the MARSHA, such as 2—which would have classified 72% of the sample as victims—the MARSHA-C would have an overall accuracy of 76% (*i.e.,* 10 percentage points lower) due to reduced sensitivity. However, a result of using the cutpoint of > 5 is that the MARSHA-C is a tool for identifying those youth who have experienced at least 5 acts of DA as opposed to fewer. A third limitation is that, with the exception of the female, heterosexual, and older respondent subgroups, demographic subsamples were too small for adequately powered tests of sensitivity and specificity. Additional research that assesses the accuracy of the MARSHA-C with sufficiently powered demographic subgroups may benefit the field. Fourth, being able to read English was an eligibility criterion. Additional psychometric research will be needed to assess the MARSHA-C in languages other than English. This additional research will provide a platform for expanding the utility of the MARSHA-C to screen youth across ethnic/cultural groups. Finally, future studies of this type may consider the use of item response theory (IRT) to identify items most discriminant and endorsed.

## Conclusion

The 3-item MARSHA-C is an effective tool that can be used to detect DA victimization among youth ages 11–21 years old. DA is common and can have long-lasting health effects. A brief screening tool can enhance pediatric health providers’ capacity to identify youth who can use additional support, referrals to helping resources and preventive guidance.

## Data Availability

The dataset supporting the conclusions of this article is in the process of being submitted to the ICPSR repository, under project number NACDJ_NIJ-130121. In addition, the datasets used and/or analysed during the current study are available from the corresponding author on reasonable request.

## Abbreviations

AUC: Area under the curve
DA: Dating abuse
MARSHA: Measure of Adolescent Relationship Harassment and Abuse
MARSHA-C: Clinical screening version of the MARSHA
ROC: Receiver operator characteristic
U.S: United States
